# Mechanistic insights and molecular therapy for neuroendocrine prostate cancer

**DOI:** 10.3389/fonc.2026.1806742

**Published:** 2026-04-22

**Authors:** Yichen Niu, Senyu Zhang, Shouyi Zhang, Jun Tao, Yuyan Zhu

**Affiliations:** 1 National Clinical Research Center for Laboratory Medicine, Department of Laboratory Medicine, The First Hospital of China Medical University, Shenyang, China; 2 Department of Urology, First Affiliated Hospital of China Medical University, Shenyang, China; 3 Department of Urology, Cancer Hospital of China Medical University, Cancer Hospital of Dalian University of Technology, Liaoning Cancer Hospital and Institute, Shenyang, Liaoning, China; 4 Department of Neurosurgery, First Affiliated Hospital of China Medical University, Shenyang, China

**Keywords:** epigenetic alteration, neuroendocrine differentiation, prostate cancer, targeted therapy, transcriptional regulation

## Abstract

Neuroendocrine prostate cancer (NEPC) is a highly aggressive subtype of prostate cancer with a poor prognosis, strongly associated with androgen deprivation therapy. Recent studies have revealed that neuroendocrine transdifferentiation in NEPC is influenced by both transcription factor activity and epigenetic regulation. This review systematically discusses the origin hypotheses, molecular subtypes, driving mechanisms, and therapeutic strategies of NEPC, focusing on the critical roles of transcription factors and epigenetic modifications in lineage plasticity. Furthermore, we summarize the latest advancements in targeted therapies. By integrating multiomics data and preclinical models, this review provides a theoretical foundation for precision treatment of NEPC.

## 1 Introduction

Prostate cancer (PCa) is one of the most common malignant tumors worldwide and a leading cause of cancer-related death (1). As a highly heterogeneous disease, prostate cancer often evolves into multiple subgroups, from the initial stage through treatment, to adapt to environmental pressures and maintain survival. Under certain epigenetic conditions, some PCa subgroups exhibit strong plasticity, high invasiveness, and lethality. Among these, neuroendocrine prostate cancer (NEPC) is the most malignant subtype, with the worst prognosis.

NEPC typically arises from adenocarcinoma following androgen deprivation therapy (ADT) or androgen receptor pathway inhibitor (APRI) treatment (2–4), and is defined as t-NEPC. Compared with well-differentiated or moderately differentiated tumors, poorly differentiated tumors are more likely to transition to NEPC (5). A small proportion of patients present with *de novo* NEPC, i.e., newly diagnosed NEPC with neuroendocrine features at initial diagnosis. Studies have shown significant differences between *de novo* NEPC and t-NEPC in terms of clinical presentation and prognosis. The median overall survival (OS) for *de novo* NEPC patients was 16.8 months, significantly shorter than the 53.5 months for t-NEPC patients (P = 0.043). Large-scale analysis of prostate cancer metastatic tissue revealed that castration-resistant prostate cancer (CRPC) primarily involves bone metastasis (31% CRPC vs. 2% NEPC), whereas NEPC predominantly involves visceral metastasis (e.g., liver metastasis), followed by lymph node metastasis (6). NEPC frequently metastasizes to visceral organs and exhibits only a transient response to chemotherapy, with most patients surviving less than 1 year (7). Currently, the primary treatment for NEPC is systemic chemotherapy combined with radiotherapy or surgery for local palliative treatment of clinically localized or metastatic disease (8). Although some patients may benefit from these treatments, the rapid progression and incurability of NEPC pose significant challenges for urologists, and the limited efficacy of existing chemotherapy regimens necessitates the exploration of novel therapeutic strategies.

In recent years, the application of next-generation sequencing technology has provided new insights into the unique molecular characteristics and evolutionary processes of NEPC, paving the way for the identification of its driving factors. In this review, we introduce the classification of NEPC from the perspectives of pathological histology and molecular subtypes. Based on epigenetic changes and genetic background, we summarize lineage plasticity, the driving mechanisms of transdifferentiation, and the formation process of the neuroendocrine phenotype in prostate cancer. By deepening our understanding of the pathogenesis of NEPC and its adaptive survival strategies, we hope to provide more directions and approaches for its treatment.

## 2 Origin and biological characteristics of NEPC

**Figure 1:**
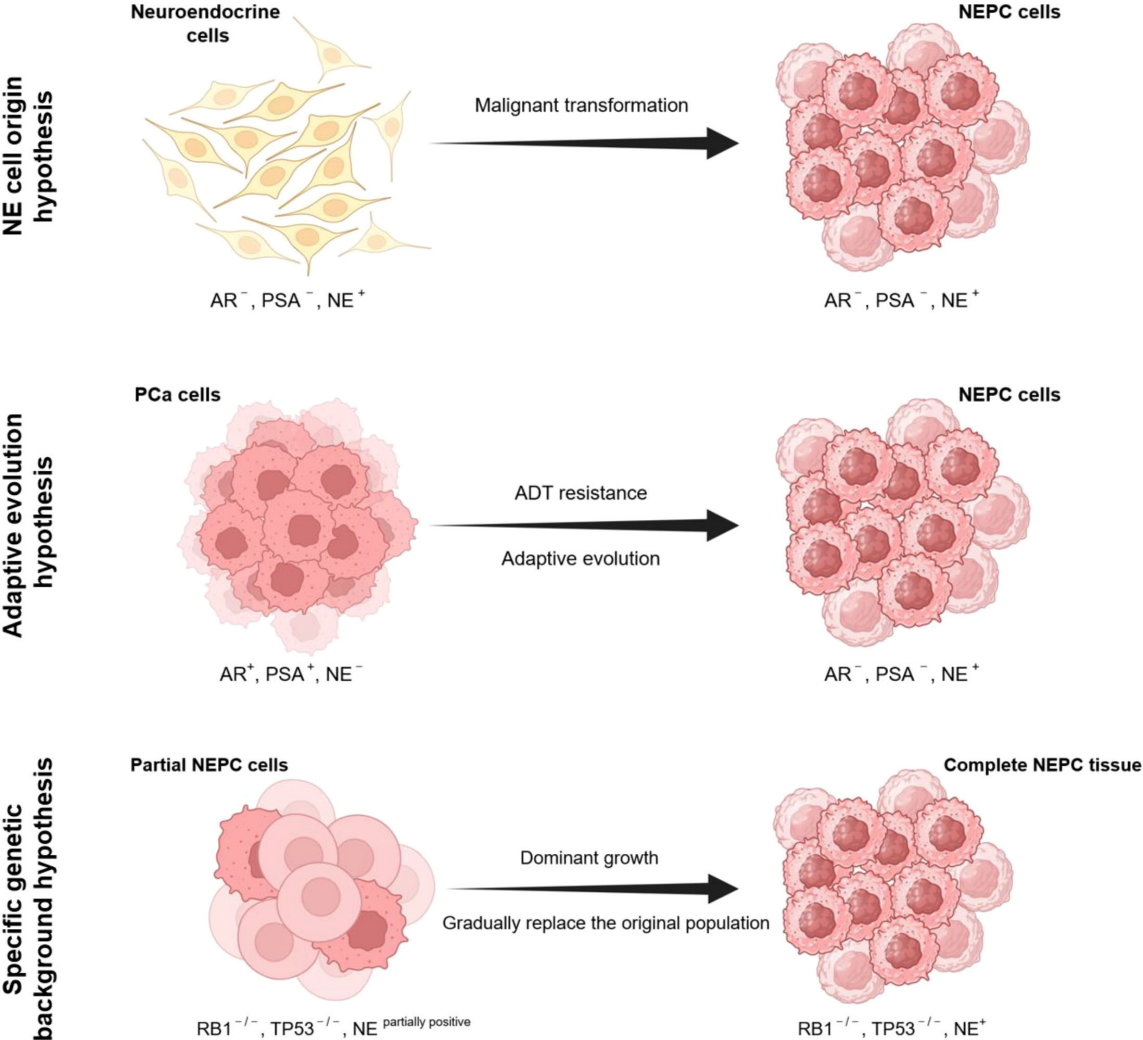
Schematic diagram of the NEPC origin hypotheses. From top to bottom, there are NE cell origin, adaptive evolution, and specific genetic background hypotheses.

NEPC is a highly aggressive and metastatic subtype of prostate cancer. Based on its clinical characteristics, it can be classified into two primary categories: (1) Primary NEPC: This subtype presents with a neuroendocrine phenotype at initial diagnosis and accounts for less than 2% of all prostate cancers. (2) Treatment-related NEPC (t-NEPC): This typically occurs after treatment for CRPC with androgen deprivation or AR inhibitor therapy, with an incidence rate of approximately 10%–17%. The median survival after diagnosis is less than 7 months. A meta-analysis based on multiple studies revealed that the incidence of dual *RB1* and *TP53* gene alterations was significantly greater in primary NEPC patients than in t-NEPC patients (9), suggesting that the two may have different molecular origins and evolutionary pathways. The origin of t-NEPC is largely believed to be associated with the adaptive evolution of tumor cells under drug pressure. The origin of primary NEPC remains controversial, with most researchers suggesting that it is associated with specific genomic alterations, while another hypothesis proposes that NEPC cells share the same origin as normal neuroendocrine (NE) cells (10) (**Figure 1**).

Initially, most neuroendocrine tumors were thought to originate from migrating neural crest cells (11). Normal neuroendocrine (NE) cells are also found in the prostate, distributed among the secretory cells of the glandular epithelium throughout the gland. These normal prostate NE cells can be divided into two types: open cells, which possess specialized microvilli extending into the glandular lumen, and closed cells, which have dendritic structures connected to epithelial and basal cells and are closely associated with afferent and efferent nerves. The primary marker for normal prostate NE cells is chromogranin A (CgA), and their secretory products include serotonin (5-HT), bombesin, neuron-specific enolase (NSE), calcitonin, and other members of the calcitonin gene family. However, they do not express androgen receptors, PSA, or Bcl-2 (12). These characteristics are partially similar to those of NEPC cells, but unlike NEPC cells, normal prostate NE cells are fully differentiated and lack proliferative capacity. In contrast, NEPC cells exhibit undifferentiated characteristics and the strong proliferative capacity typical of tumor cells. Because of their robust neuroendocrine properties, NEPC cells can secrete certain growth factors, such as insulin-like growth factor-1 (IGF-1), keratinocyte growth factor (KGF), interleukin-6 (IL-6), and forskolin, even without hormones. These factors functionally replace DHT and activate the androgen receptor (AR) signaling pathway (13–15). The outcome of NEPC is often associated with multiorgan metastasis and poor survival prognosis, which may be related to the enhanced proliferation and invasiveness of PCa due to NE hormones (12). The NE cell origin hypothesis was initially derived from neuroblastoma; primitive neural crest cells may undergo specific mutations and acquire additional genomic alterations, leading to transformation into NE tumors (16). In the prostate, a study using a genetically engineered mouse model provided experimental support for the NE cell origin hypothesis (17). Inducing SV40 T-Ag expression in a subpopulation of neuroendocrine cells in mouse prostate lobes resulted in the migration of NEPC tumor cells expressing SV40 T-Ag and neuroendocrine markers (SYP and CHGA) to regional lymph nodes and resistance to castration after a period of time. This study suggests that prostate neuroendocrine cell populations can undergo malignant transformation.

Based on multiomics analysis of clinical patient samples and studies using genetically engineered mice or organoid models, researchers have gained a clearer understanding of the origin of NEPC. Strong evidence suggests that specific genomic alterations are closely associated with the development of neuroendocrine phenotypes. For example, knocking out the TP53 and RB1 genes in normal human prostate epithelial cells can facilitate the transition from the epithelial to the neuroendocrine lineage (18). Similarly, specifically inducing the loss of PTEN, TP53, and RB1 in prostate tubular cells in mouse models can also result in neuroendocrine prostate cancer (19). Additionally, genomic data from clinical patients support this view (20, 21). Notably, genetically engineered organoids and mouse models directly resulted in the generation of scattered NEPC cells with *de novo* deletions of RB1 and TP53, which gradually replaced the original tissue cells over time, similar to *de novo*-occurring NEPC. These findings suggest that genomic instability in a specific genetic background plays a key role in the generation of NEPC cells.

Although most patients initially benefit from ADT, the therapeutic effects of ADT are limited, and most patients eventually develop CRPC. To adapt and survive under therapeutic pressure, tumor cells often undergo lineage plasticity, transforming from one cell type to another (22, 23). Reports of small cell neuroendocrine prostate cancer emerging during treatment are common in both clinical cases and cell and mouse models (3, 6, 24–27). For example, approximately 50% of NEPC patients exhibit prostate cancer-specific TMPRSS2-ERG gene rearrangements, a proportion similar to the incidence of prostate adenocarcinoma (PRAD) (28–30). In the androgen-sensitive LNCaP prostate cancer cell line, the acquisition of ADT resistance further increases lineage plasticity (21, 31). In genetically engineered mouse models, abiraterone treatment leads to tumor progression to a small cell/neuroendocrine-like phenotype (27). This evidence suggests that NEPC and adenocarcinoma share a common clonal origin.

## 3 Epigenetic characteristics of NEPC and CRPC

**Figure 2:**
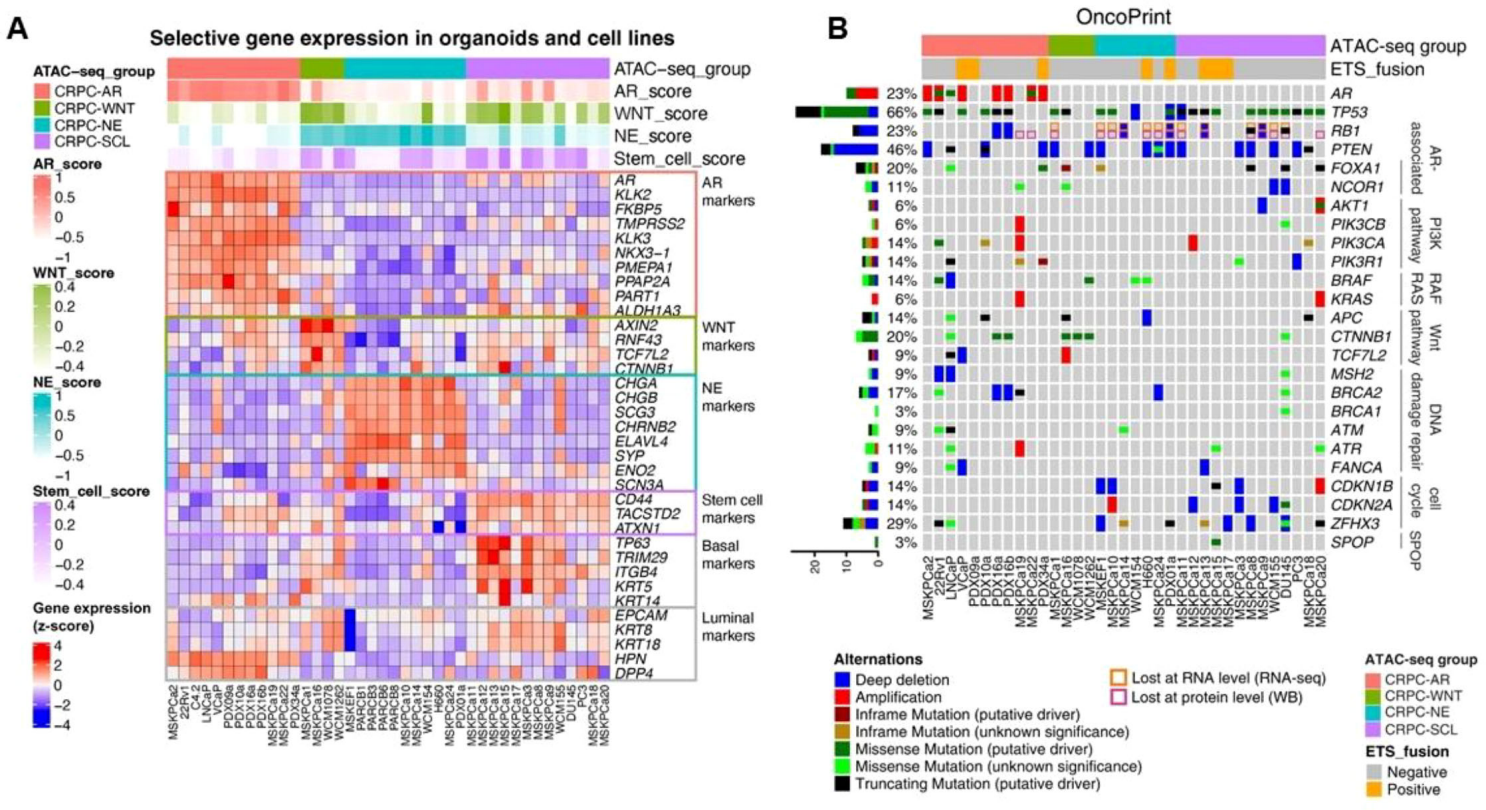
Genomic characterization of different CRPC subtypes via ATAC-seq and DNA sequencing (32). **(A)** A heatmap shows the relative expression of subtype-specific marker genes and basal/lumen genes in all the samples. **(B)** DNA sequencing data show the genomic alterations in all the samples.

In the hypothesis of NEPC origin, adaptive evolution under ADT pressure is thought to account for the vast majority of NEPC production. This process not only relies on AR inactivation characteristics in NEPC but is also potentially linked to several features associated with rapidly progressing CRPC, including low PSA levels, paraneoplastic syndromes, visceral metastases (such as liver and brain), and elevated serum levels of neuroendocrine markers such as CgA, NSE, and gastrin-releasing peptide (GRP) (8). These features not only assist in the diagnosis of NEPC but also suggest the presence of neuroendocrine subtypes in CRPC. A recent study conducted an in-depth analysis of 22 prostate cancer organoids, 6 xenografts, and 12 cell lines via ATAC-seq, RNA-seq, and DNA sequencing techniques and divided CRPC into four molecular subtypes based on their gene expression characteristics (**Figures 2A, B**) (32):

1. CRPC-AR is enriched for the AR signature. This subtype is characterized by AR and FOXA1 as the main transcription factors (TFs) and is frequently associated with AR gene amplifications, activating point mutations, and splice variants such as AR-V7 (32). Additionally, known AR coactivators (such as FOXA1, NCOR1/2, and ZBTB16) are also altered (6).
2. CRPC-WNT, which is enriched for Wnt signaling, exhibits basal-like and basal stem cell-like properties, with enrichment in fibroblast growth factor receptor (FGFR) signaling and selective expression of FGF ligands and receptors. TCF/LEF is the key transcription factor in this subtype, with TCF4 being the most significantly expressed.
3. CRPC-NE, which is enriched for the NE signature, also exhibits basal and basal stem cell-like properties, accompanied by deep loss of RB1. Neurogenic differentiation factor 1 (NEUROD1) and achaete-scute homologue 1 (ASCL1) are the major transcription factors in this subtype (32). Somatic alterations of the AR gene in this subtype are not significant, and the expression of AR-V7 and wild-type AR is low (6), which may be attributed to clonal selection of tumor cell subpopulations resistant to ADT pressure.
4. CRPC-SCLC, which consists of stem cell-like cells, also exhibits basal and basal stem cell-like properties. The cancer stem cell markers CD44 and TACSTD2 (TROP2A) are highly expressed in this subtype, accompanied by FGFR signaling enrichment and selective expression of FGF ligands and receptors. The AP-1 family is the main transcription factor in this subtype, with FOSL1 expression being the most prominent.

Among these four subtypes, TP53 mutations are the most common (66%), followed by RB1 (20%) and PTEN (43%) mutations. Notably, the loss of TP53 and RB1 results in similar basal cell-like and basal stem cell-like properties in different subtypes of CRPC, indicating that cells with the same genetic background exhibit similar plasticity. NEPC and CRPC share similar genetic alterations in RB1 and TP53. Given this shared plasticity, differences in key transcription factors between subtypes are the decisive factors leading to distinct evolutionary programs. In this study, the key transcription factors of CRPC-NE were NEUROD1 and ASCL1. However, other studies have indicated that the expression of key transcription factors may differ depending on genomic alterations. For example, the overexpression of c-Myc, myrAKT1, and BCL-2, in conjunction with the knockout of TP53 and RB1 in normal human prostate epithelial cells, has been shown to induce transformation from the epithelial lineage to the neuroendocrine lineage. During this process, the function and activity of transcription factors containing LIM domains (such as LHX2, ISL1, LHX3, and LHX1) are significantly improved (18). A similar phenomenon has been validated in genetically engineered mouse models. By specifically inducing PTEN, TP53, and RB1 deletions in prostate luminal cells, researchers successfully constructed a neuroendocrine prostate cancer model and observed the complete evolutionary time series of tumors via single-cell sequencing. One study revealed that the pioneer transcription factor FOXA2 drives the transition of prostate cancer from adenocarcinoma to the neuroendocrine lineage and directly activates the KIT pathway (19). In addition to FOXA2, several cell fate-determining molecules that drive development and organogenesis, such as FOXA1 (33), ERG (34), N-Myc (35), BRN2 (36), EZH2 (37), ONECUT2 (38), and REST (39), have been shown to be involved in NEPC development processes. These transcription factors are closely linked to the acquisition of luminal cell lineage transdifferentiation in prostate cancer.

It follows that a variety of NE TFs (neuroendocrine transcription factors) can maintain the NE state in NEPC, as has been demonstrated in different neuroendocrine carcinomas (NECs) (40). Interestingly, different NECs share a common landscape of DNA-accessible regions and are even similar in pathological features, which could result from the activation of common transcriptional regulators, particularly between NEPC and SCLC. Since different NE TFs play roles in driving neuroendocrine processes, how do they differ from one another? Is it possible that different TFs predominate in subpopulations with distinct characteristics? This phenomenon was also observed in the present study: ASCL1 and NEUROD1 were expressed in distinct subsets of NEPC patient tissues and were mutually exclusive in the NEPC cohort. Further results revealed the existence of distinct genetic clones associated with each of the two NEPC epigenetic subtypes in this patient, likely derived from a common ancestor given their substantial CNV profile overlap. Although NEPC subtypes have not been fully explored in large clinical cohorts, these results suggest that NEPC may give rise to multiple subtypes with different functions and characteristics due to variations in key transcription factors across different genomic alterations.

## 4 Pathology and histological classification of NEPC

**Figure 3:**
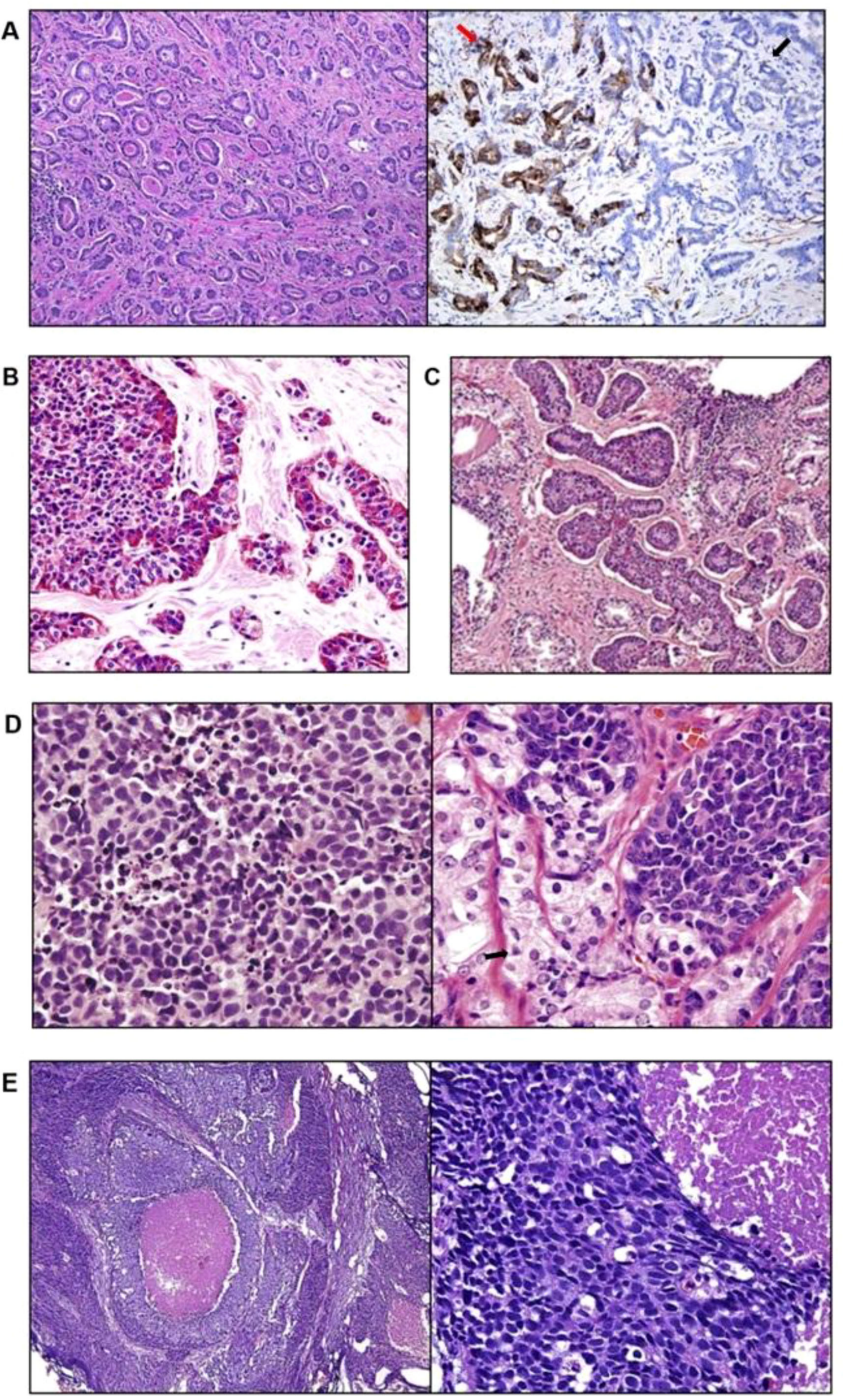
Histological morphological features of NEPC under different pathological classifications (42, 43). **(A)** Prostate adenocarcinoma lattice Gleason score 3 + 3 = 6; synaptophysin-positive areas (red arrows) could not be distinguished from synaptophysin-negative areas (black arrows) in terms of gland morphology. **(B)** Adenocarcinoma of the prostate with Paneth cell-like NE granules. **(C)** Carcinoid tumor with nests of cells. **(D)** Small cell carcinoma of the left prostate, mixed adenocarcinoma (black arrow), and small cell carcinoma (white arrow) of the right prostate. **(E)** Large fine NE carcinoma of the prostate with prominent nucleoli, large nest cells, geographic necrosis, and cells with abundant cytoplasm.

In addition to molecular classification, pathological classification is commonly used in clinical practice for prostate cancer. According to the latest WHO classification of prostate cancer, CRPC that exhibits complete (or partial) neuroendocrine differentiation is termed t-NEPC. NEPC is described as having a range of histological features, from a pure neuroendocrine morphology (most commonly small cell carcinoma and occasionally large cell neuroendocrine carcinoma) to mixed neuroendocrine tumors with poorly differentiated adenocarcinoma components. The histological and immunohistochemical (IHC) features of t-NEPC are highly similar to those of primary prostate small cell carcinoma (41). Combined with the classification methods of American surgical pathology based on morphology (42), NEPC can be roughly categorized as follows: common prostate cancer with NE differentiation, adenocarcinomas with Paneth cell NE differentiation, carcinoid tumors, small cell carcinoma, and large cell NE carcinoma. Here, we describe the classification of NEPC based on histological and cytological features, IHC features, and clinical features (**Figure 3**; **Table 1**):

**Table 1:** Pathological classification and characteristics of neuroendocrine prostate cancers.

Types of pathology	Morphological characteristics	IHC markers	Clinical features
Typical prostate cancer with NE differentiation	Scattered argentaffin cells	PSA^+^, NE^+^, Ki67^+^	No significant effect on prognosis
Paneth cell-like NE adenocarcinoma	Eosinophilic granule cells	PSA^+^, NE^+^, Ki67^+^	Unclear clinical significance
Carcinoid tumor	Nests with uniform nuclei	PSA^-^, NE^+^, Ki67 ^low^	Locally advanced and the prognosis is good
Small cell carcinoma	High nuclear to cytoplasmic ratio, apoptotic bodies	PSA^-^, NE^+^, Ki67^high^	Visceral metastasis with short survival time
Large cell NE carcinoma	Large nest cells and geographic necrosis	PSA^-^, NE^+^, Ki67^high^	The average survival time was 7 months

NE, neuroendocrine; PSA, prostate-specific antigen; and Ki67, a proliferation marker encoded by the gene *MKI67*.

1. Typical prostate cancer with NE differentiation: Argentaffin cells are present in prostate adenocarcinoma and can appear as dispersed NE cells on IHC (**Figure 3A**). NE cells are positive for PSA to different degrees; therefore, these cells are recognized as prostate cancers with NE differentiation. Most studies have shown that NE differentiation in typical adenocarcinoma does not affect patient outcomes; therefore, American surgical pathology does not recommend the routine use of IHC staining to detect any NE differentiation in morphologically typical primary prostate adenocarcinoma (42). This means that routine IHC staining does not effectively distinguish typical prostate cancer from NEPC, so morphology remains the gold standard for diagnosing NEPC.
2. Adenocarcinoma with Paneth cell NE differentiation: Under routine optical microscopy, some cells have prominent eosinophilic cytoplasmic granules. Moreover, they are positive for chromogranin and contain neurosecretory granules. This phenomenon is referred to as Paneth cell-like change, which is currently used to describe unique eosinophilic NE cells. Paneth cell-like NE differentiation in prostate adenocarcinoma appears as patchy isolated cells or as diffuse involvement of glands or nests (**Figure 3B**). In some cases, a series of Paneth cell-like cells with eosinophilic granules can be observed adjacent to amphophilic cytoplasm lacking granules. Both cell types show diffuse staining for NE markers, but the cellular morphology is unremarkable, and the lesions are also limited. Currently, the clinical significance of Paneth cell-like NE differentiation remains unclear, but NE differentiation can be confirmed by IHC staining.
3. Carcinoid tumors: True carcinoid tumors of the prostate are exceedingly rare. Histologically, they are characterized by a “carcinoid” appearance, with a nested structure and uniform nuclei (**Figure 3C**). The clinical and pathological features of prostate carcinoid include no close association with accompanying prostate adenocarcinoma; positive NE markers and negative PSA on IHC; and origination from the prostate parenchyma. Prostate carcinoid tumors are well-differentiated NE tumors that often present as locally advanced disease and have a good prognosis, although regional lymph node metastasis can occur in some cases. In contrast, carcinoid adenocarcinoma is a distinct type of tumor with manifestations similar to those of common prostate cancer, such as PSA expression. Carcinoid adenocarcinoma shows NE differentiation on IHC, and most cases do not present with carcinoid syndrome (42). Only a few patients produce sufficient adrenocorticotrophic hormone, leading to Cushing syndrome (43).
4. Small cell carcinoma: Small cell carcinoma is a high-grade tumor with typical morphological features, including a lack of prominent nucleoli, nuclear molding, fragility and crushing artifacts, a high nuclear–cytoplasmic ratio, blurred cell borders, a high mitotic rate, and apoptotic bodies (**Figure 3D**). In rare cases, patients may present with *de novo* prostate small cell carcinoma, which is a poorly differentiated neuroendocrine cancer with histology similar to that of small cell lung cancer (SCLC) and other small cell carcinomas. According to a study of 95 patients with small cell prostate cancer, the median PSA was only 4.0 ng/mL, and the Gleason score of adenocarcinomas was ≥8 in 85% of these patients. Among the 61 cases (64%), small cell carcinoma presented a typical “oat cell” morphology, whereas the remainder presented “intermediate cell” variants. The majority (88%) of small cell carcinomas were positive for at least one neuroendocrine marker (44). Neurosecretory granules are also present in these small cell carcinomas (44). In another study of 18 cases of prostate small cell carcinoma, 17%–25% of the cases were PSA positive, and 90% presented one or more neuroendocrine markers (synaptophysin, chromogranin A, and CD56). p63 and high-molecular-weight cytokeratin are positive in 24% and 35% of cases, respectively, while these markers are usually negative in prostate cancer (45). Similar to PRAD, nearly half of prostate small cell carcinoma cases are positive for TMPRSS2–ERG gene fusion according to FISH staining (28). Additionally, patients with small cell carcinoma frequently have visceral metastasis, but paraneoplastic syndrome is less common.
5. Large-cell NE carcinoma: Prostate large-cell carcinoma is also a high-grade tumor characterized by large nests with peripheral palisading and regional necrosis. Its cytological features include those of non-small cell carcinomas (prominent nucleoli, vesicular clumps of chromatin, large cell size, and abundant cytoplasm) associated with high mitosis rates and extensive staining of at least one NE marker (synaptophysin, chromogranin, and CD56) (**Figure 3E**). Focal or absent PSA and PSAP expression is common, and prostate markers are generally negative. Large-cell NE carcinoma tends to spread rapidly, so the mean survival of patients is 7 months (46).

Although NEPC is diverse in terms of pathological type, its pathological subtypes are not clearly distinguished in terms of molecular type. Current research on NEPC has focused primarily on small cell neuroendocrine carcinoma, so the generation mechanism and driving factors of other pathological subtypes of NEPC warrant further exploration.

## 5 Lineage plasticity in NEPC

NEPC rarely occurs *de novo*, and newly diagnosed NEPC accounts for less than 2% of all prostate cancer cases (47). However, 10%–17% of patients with CRPC or those with ADT-treated adenocarcinoma may develop NEPC (3, 4). Increasing evidence suggests that lineage plasticity is one of the main mechanisms by which prostate tumors escape from ADT and androgen receptor signaling inhibitors (ARSIs) (19, 21, 31, 48–50). Lineage plasticity is a biological process that initially occurs during normal development and later becomes a mechanism by which cells adapt to their environment, escape stress, or repair tissue to promote survival. This plasticity can manifest as reversible or irreversible changes in the “identity” of cells, leading to different morphologies, phenotypes, or epigenetic states. Lineage plasticity not only depends on tumor heterogeneity but also further drives the enhancement of this heterogeneity, thus increasing the diversity of tumor cell subpopulations and being closely related to treatment resistance and metastasis. Drug resistance caused by lineage plasticity mainly occurs in treatments that target the main cell growth programs and lineage guiding factors, such as BRAF-mutated melanoma, EGFR-mutated lung cancer, and AR-driven prostate cancer. These treatments usually work by inhibiting essential signaling pathways for cell survival. Under therapeutic pressure, the adaptive survival mechanisms of tumor cells can be explained from two perspectives: (1) the self-evolution of tumor cells under survival stress and (2) the selective cloning of tumor cells because drug-resistant subsets are naturally heterogeneous. These two perspectives represent the roles of epigenetics and classical genetics in tumor progression, respectively. Next, we discuss potential factors driving the development of NEPC from these two perspectives.

### 5.1 Plasticity driven by genetic background

Multiple studies have identified recurrent somatic mutations, copy number changes, and oncogenic DNA rearrangements in primary prostate cancer, including point mutations in *SPOP*, *FOXA1*, and *TP53*; copy number alterations in *MYC*, *RB1*, *PTEN*, and *CHD1*; and E26 transcription factor (ETS) fusions (51–57). These genomic changes often co-occur, and the overlap of mutations between genes can alter cell behavior. As previously mentioned, NEPC often evolves from PRAD, with which it shares some genetic similarities. Current research suggests that key genomic events influencing NEPC development include the loss of *RB1*, as well as the mutation or deletion of *TP53* and *PTEN*.

*PTEN* and *TP53* are tumor suppressor genes and among the most frequently inactivated or mutated genes in human cancers. *TP53* primarily regulates gene expression programs, leading to cell cycle arrest or apoptosis. *TP53* mutations or deletions occur in 66.7% of NEPC cases and 31.4% of CRPC cases and are commonly associated with metastasis and androgen-independent phenotypes (6, 58, 59). *PTEN* can regulate the stability of p53, and p53 can increase *PTEN* transcription (60–62). Conditional inactivation of *TP53* in the mouse prostate does not produce tumor phenotypes, whereas complete loss of *PTEN* leads to nonlethal invasive prostate cancer after a long latency period. The combined loss of *PTEN* and *TP53* induces invasive prostate cancer as early as 2 weeks after puberty and is often fatal by 7 months of age (63). Furthermore, researchers have observed the same androgen deprivation-resistant cell populations in tumors from PTENPE-/- and RB1PE-/-mice subjected to both castration and noncastration protocols, suggesting that ADT resistance-associated tumor cell populations are present at an early stage of prostate cancer with both *PTEN* and *RB1* loss (64). These findings also suggest that tumor cells are prone to multiple evolutionary pathways under this genetic background.

One of the major functions of RB1 is to control the G1–S phase of the cell cycle, and its inactivation leads to uncontrolled cell proliferation (65). Approximately 70% of NEPC cases and 10%–30% of CRPC cases exhibit RB1 biallelic loss or functional loss, which is significantly associated with poor patient prognosis (66, 67). In metastatic CRPC (mCRPC), RB1 loss is more frequent (56%) than in hormone-sensitive prostate cancer (HSPC) (35%) (68), suggesting that RB1 loss may provide a survival advantage to prostate cancer cells. Another study confirmed that RB1 loss is an early driver of lineage plasticity, promoting the emergence of androgen-independent and NEPC tumor phenotypes (21, 31). Indeed, single loss of RB1 or TP53 in mouse models leads to only prostatic intraepithelial neoplasia (PIN), whereas the combined loss of RB1 and TP53 results in metastatic cancer, with metastases to the lungs, liver, adrenal glands, and regional lymph nodes (64). Although metastatic cancer resulting from the combined loss of RB1 and TP53 differs in the site of metastasis from that in human prostate cancer (commonly bone metastasis), we can also infer from this gene editing that single genetic defects alone do not tend to produce fatal tumors. Similarly, in human cancers, RB1 loss co-occurring with TP53 mutations is common in primary small cell prostate cancer and lung cancer, which may contribute to the pathogenesis of small cell cancer (64, 69). With respect to the specific mechanisms involved in tumor progression, RB1 loss not only promotes the growth of NE-like cells with reduced AR signaling but may also stimulate lineage reprogramming by upregulating SOX2 expression in combination with TP53 loss (21). The chromatin binding and transcriptional activity of E2F1 in RB-deficient CRPC are highly dependent on LSD1/KDM1A, and RB inactivation makes CRPC tumors sensitive to LSD1 inhibitors (70). Additionally, RB1 loss can activate the master regulator transcription factor ONECUT2 (OC2) in mCRPC. The upregulation of OC2 can drive metastasis and lineage plasticity, especially in neuroendocrine differentiation. Moreover, OC2 expression can also downregulate TP53 transcription and induce RB1 inactivation through phosphorylation (71).

### 5.2 Lineage transformation driven by transcription factors and epigenetic regulators

**Figure 4:**
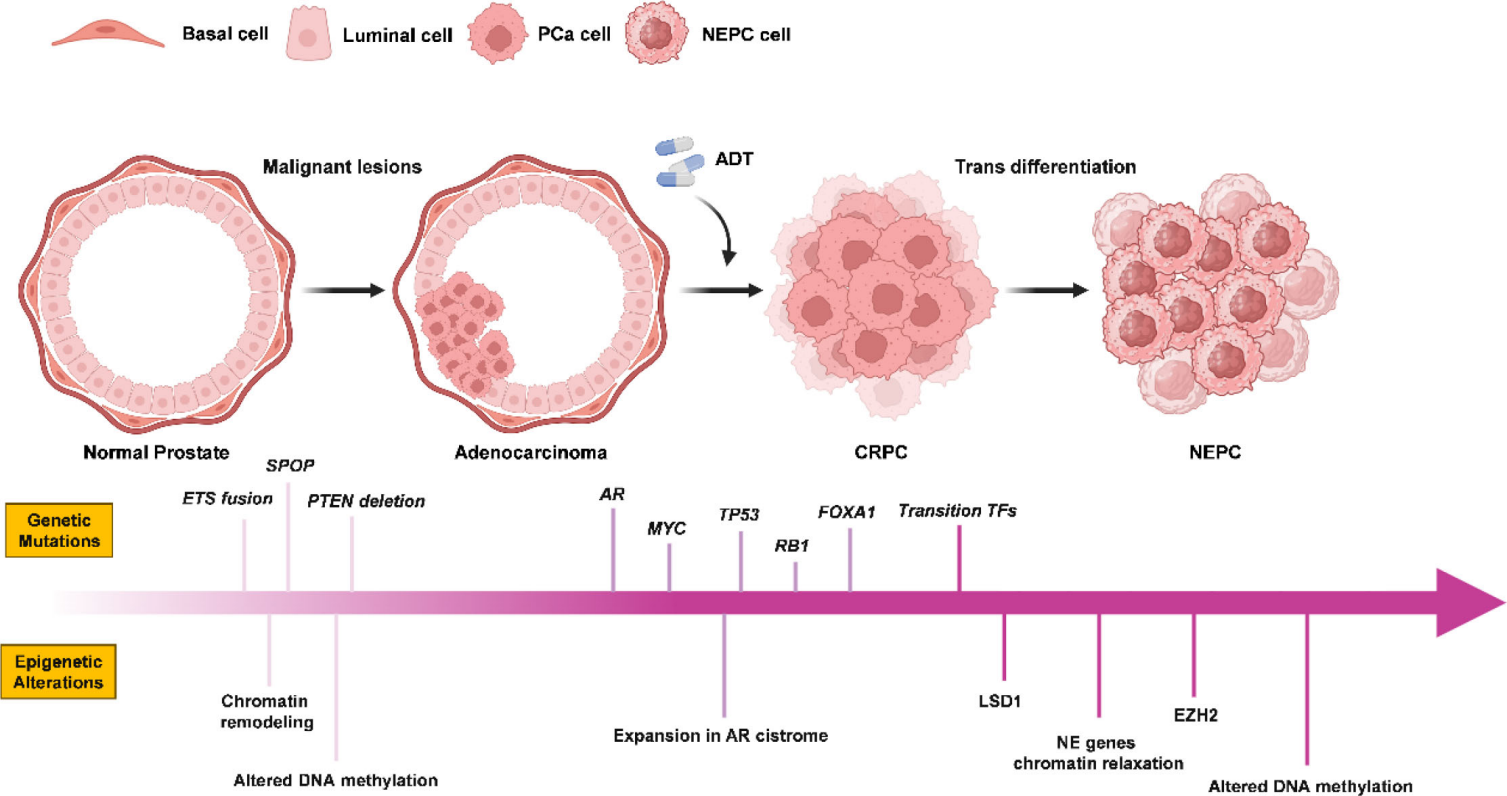
Diagram of the evolution of the NEPC. From left to right are the normal prostate, prostate adenocarcinoma, CRPC, and NEPC. Gene mutations during the evolution of NEPC are above the long arrow, and epigenetic changes are below the long arrow.

Previously, we discussed the role of genetic background in determining cell identity and characteristics. The plasticity of cells changes under certain genomic alterations, and can be understood as a cellular state in which cell lineages are highly susceptible to change. Currently, epigenetics plays a key role in lineage transformation, resulting in the acquisition of stem-like cell properties and changes in developmental processes and differentiation (72). Genome-wide analyses have shown that DNA methylation patterns can effectively distinguish NEPC from CRPC. Functional enrichment analysis of differentially methylated genes revealed epigenetic dysregulation pathways involved in NEPC progression, including neuronal and intercellular adhesion, development, epithelial–mesenchymal transition (EMT) and stem cell programs (6). Changes in DNA methylation ultimately affect gene expression; in fact, transcription factors play a key role in driving lineage transformation and are recognized as key regulators of cell fate reprogramming (73). During tumorigenesis, some molecules that drive development and organogenesis, and determine cell fate, such as FOXA1 (33), ERG (34), N-Myc (35), BRN2 (36), ONECUT2 (38), and REST (39), are involved in cancer lineage plasticity. These transcription factors are closely related to the acquisition of lineage plasticity in prostate cancer luminal cells. Next, we describe the role of these transcription factors in lineage transformation in NEPC (**Figure 4**).

1. FOXA family proteins are well-known pioneer factors that can displace adaptor histones on nucleosomes, thereby opening compact chromatin structures and increasing the accessibility of other transcription factors (74). FOXA1 is crucial for the normal development of various endodermal-derived organs, including the prostate (75, 76). In prostate adenocarcinoma, FOXA1 serves as a key pioneer factor for AR, and the FOXA1/AR signaling axis is essential for the development of this tumor subtype (77). FOXA1 is frequently mutated or altered in CRPC, and these gain-of-function alterations can promote resistance to AR inhibitors (ARSi) (33). In NEPC, despite the loss of dependence on AR, FOXA1 expression is maintained and is required for proliferation and the expression of NE lineage-defining genes. Ectopic expression of the NE lineage TFs ASCL1 and NKX2–1 in PRAD cells reprograms FOXA1 to bind to NE regulatory elements, inducing enhancer activity (78). FOXA2 appears to play a role in the initiation phase of tumor cells in multiple lineages. In luminal androgen receptor-positive prostate cancer cells, FOXA2 expression alone does not determine the fate of lineage reprogramming; it requires cooperation with other factors, such as JUN (79). FOXA2 expression is induced by androgen deprivation during the progression of prostate cancer and plays a key role in mediating the transition from the prostate lumen to the NE lineage and KIT signaling activation in NEPC (19). In fact, FOXA2 overexpression can drive the generation of three subtypes—CRPC-SCL, NE, and WNT—by binding to enhancers. The chromatin binding of both FOXA1 and FOXA2 is regulated by demethylation mediated by lysine-specific demethylase 1 (LSD1) or KDM1A (79, 80). These findings suggest that the effects produced by the FOXA family are closely related to several key chromatin-associated proteins, which may become key targets that indirectly affect the function and lineage transformation of the FOXA family.
2. ASCL1 is a neurogenic transcription factor that drives tumor proliferation and the expression of downstream neuronal and neuroendocrine lineage pathways. ASCL1, a marker of NE differentiation that may be involved in the expression of NE-related genes, is upregulated in NEPC (81–83). In a previous study, ASCL1 was found to play a crucial role in driving neuroendocrine lineage reprogramming in prostate cancer by suppressing AR signaling and promoting neuroendocrine transformation, while also increasing the accessibility of the CEACAM5 (CEA) core promoter to chromatin. CEA, a membrane surface carcinoembryonic antigen, is highly expressed in some subgroups of NEPC, offering a potential avenue for targeted therapy (84). Furthermore, ASCL1 overexpression significantly enhances the progression from CRPC to NEPC by resisting ferroptosis (85).
3. EZH2 is a histone methyltransferase and a catalytic component of polycomb repressive complex 2 (PRC2). EZH2 mediates the trimethylation of histone H3 lysine-27 (H3K27me3) and is required for PRC2-mediated gene silencing (86). EZH2 is associated with invasive progression in prostate cancer and is more enriched in NEPC than in adenocarcinoma (87–89). Compared with other prostate cancer cell types, EZH2 inhibitors are more effective at reducing the activity of NEPC cells (NCI-H660), accompanied by the downregulation of NEPC-related genes, including NCAM (CD56), MYCN, and PEG10 (6). These findings suggest that EZH2 promotes NE differentiation through epigenetic regulation and that the epigenome plays a key role in the emergence and maintenance of the NEPC phenotype.
4. AURKA and MYCN gene amplification is present in approximately 65% of primary prostate adenocarcinoma samples that subsequently develop NEPC after ADT, and 90% of these cases exhibit metastasis (90). Another study found that AURKA and MYCN were significantly overexpressed and amplified in 40% of NEPC cases and 5% of prostate cancer cases and that they synergistically induced the NE phenotype in prostate cells (90). AURKA and MYCN are also markers of NE differentiation and key molecules regulating NE differentiation. N-myc (MYCN), a member of the Myc family of basic helix-loop-helix zipper (bHLHZ) transcription factors, serves as a central regulator of many important cellular processes and is involved in the development of the nervous system. It is widely known for its classical oncogenic activity and association with human neuroblastoma. N-myc is generally not expressed in the prostate but is highly expressed in many neurogenic and neuroendocrine tumors (91, 92), and it is significantly overexpressed in 40% of NEPC patients and 5% of PRAD patients (90). N-Myc overexpression is associated with the induction of NEPC histological phenotypes and molecular programs. In normal human prostate epithelial cells, the overexpression of N-Myc and activated AKT1 can transform them into prostate cancer cells, with some regions exhibiting NEPC histological phenotypes and molecular characteristics (93). In mouse and cell models containing AR, N-Myc binds to AR enhancers and interacts with AR in a process dependent on the catalytic activity of EZH2, ultimately inhibiting AR signaling (37). AURKA is a serine/threonine kinase that plays an important role in cell cycle regulation, and its family includes three members: AURKA, AURKB, and AURKC. Both AURKA and AURKB play important roles in mitosis and the regulation of cell division, whereas AURKC plays unique physiological roles in spermatogenesis. There are few studies on AURKC in cancer. AURKA and AURKB have been identified as oncogenes that promote tumorigenesis in various cancers, including solid tumors and hematologic malignancies (94). In human neuroblastoma, AURKA has been shown to interact with and stabilize the oncogene N-MYC (95). AURKA is frequently upregulated in NEPC tumors (6). Interestingly, the inhibition of AURKA results in synthetic lethality in the absence of RB1 and TP53. Although these two studies focused on cell models with RB1 and TP53 deletions rather than prostate cancer, they suggest that AURKA plays a key role in driving the cell cycle of these tumors (96, 97). Additionally, after AR inhibition in prostate cancer, AURKA is regulated by NK1R and collaborates with N-Myc to promote NE transdifferentiation in prostate cancer cells (98). Moreover, AURKA can reduce NKX3.1 protein levels by phosphorylation in CRPC and NEPC, driving cells toward a highly invasive carcinogenic phenotype and increasing the expression of NE markers (synaptophysin and enolase) (99).
5. OC2 is a newly discovered member of the ONECUT transcription factor family. As a transcription factor, OC2 can broadly regulate the expression of proteins involved in cell proliferation, migration, adhesion, differentiation, and cellular metabolism. In tumor tissues, it is associated with cell proliferation, angiogenesis, metastasis, and EMT (100). In prostate cancer, OC2 has been confirmed as a key transcriptional regulator in mCRPC, where it inhibits the expression and regulation of AR and FOXA1, thus promoting the development of NE characteristics (49). In regulating prostate cancer lineage plasticity, OC2 drives lineage plasticity by binding promoters to regulate the gene expression of glucocorticoid receptors (GRs, NR3C1) and the NE splicing factor SRRM4. Moreover, OC2 can also enhance genome-wide chromatin accessibility and superenhancer reprogramming (101). This phenomenon also occurs in breast cancer, where OC2 inhibits gene expression programs associated with luminal differentiation by inhibiting ER expression and activity and activates basal-like states at the gene expression level (102).
6. PEG10 is a paternally expressed imprinted gene that plays an important role in the Eutherian development system and is associated with developmental abnormalities caused by abnormal genomic imprinting (103). PEG10 is suppressed by AR in PCa, but its expression rapidly increases during ADT, helping PCa cells survive and resume proliferation under ADT stress. PEG10 promotes the invasive and proliferative phenotypes of NEPC cells. Its function is closely related to the loss of RB1 and TP53, and it plays an important role in the transdifferentiation of PCa (104).
7. Novel NE TFs: By collecting and analyzing published single-cell RNA sequencing (scRNA-seq) datasets combined with large models and algorithms, researchers have identified novel NEPC-associated transcription factors, including PHTF, LHX2, ZNF702P, NANOS1, LBX2, ZHX2, SHOX2, HOXC8, and XBP1 (105). The roles of some of these molecules in prostate cancer have been demonstrated. For example, SHOX2 is closely related to the proliferation and metastasis of prostate cancer, and the hypermethylation of circulating tumor DNA can serve as a marker for the detection of metastatic prostate cancer (106–108). The expression of HOXC8 in prostate cancer is closely associated with malignant biological behaviors such as the loss of tumor differentiation, tumor proliferation, and metastasis (109–112). XBP1 is closely related to the activation of AR signaling and c-MYC signaling (113–115). The transcriptional activity of LHX2 was significantly increased during the transformation of normal human prostate epithelial cells into neuroendocrine prostate cancer cells via gene editing (18). Although there is no solid evidence to support their role in NEPC transdifferentiation, the ability of these molecules to promote malignant biological phenotypes of prostate cancer has been demonstrated.

In summary, we can conclude that epigenetic changes in TP53 and RB1 are necessary preconditions for the occurrence of NEPC and that abnormal expression of NE-related transcription factors is a key factor driving the development of NEPC.

## 6 Therapeutic approaches based on transcription factors and epigenetic regulation

Although researchers have made significant progress in understanding the origin and progression of NEPC, the translational and clinical significance of these research findings remains unclear. A variety of therapeutic strategies have been developed for hormone-sensitive prostate cancer, including hormone therapy (116, 117), PARP inhibitors (118), immune checkpoint inhibitors (119), radiopharmaceuticals and radiotherapy (120), chemotherapy (121–123), antibody-drug conjugates (124), epigenetic regulators, and signaling pathway inhibitors (125). In comparison, clinically localized small cell prostate cancer is typically treated with chemotherapy and radiotherapy. The treatment for metastatic small cell prostate cancer is platinum-based combination chemotherapy, similar to that used for small cell lung cancer. Whether the treatment of mixed small cell carcinoma differs from that of pure small cell carcinoma depends on the clinical context. Patients with clinically aggressive metastatic mixed tumors are usually treated with ADT and chemotherapy (platinum combined with etoposide or platinum combined with taxane) (42). Clinical data indicate that the efficacy of second-line treatment is generally limited, regardless of whether the patient has primary NEPC or t-NEPC. In a multicenter retrospective study, patients who progressed after first-line platinum-based chemotherapy were treated with various second-line regimens; however, regardless of the treatment chosen, the objective response rate was low (23.5%), and the median overall survival was only 7.4 months. These findings suggest that conventional chemotherapy has limited overall efficacy for NEPC, particularly in the advanced stage, and that there is an urgent need for more effective targeted and immunotherapeutic strategies (126). The following discussion explores the possibilities of NEPC treatment from the perspectives of immunotherapy and targeted therapy.

### 6.1 Targeting epigenetic regulators

1. DNA methyltransferase (DNMT): Decitabine is a pan-DNMT inhibitor. Compared with RB1-positive CRPC, decitabine can reduce tumor growth in xenograft models derived from NEPC patients and RB1-deficient CRPC models. Moreover, DNMT inhibitors enhance the expression of B7 homologue 3 (B7-H3) through demethylation. Combined targeting of B7-H3 with DNMT inhibitors can enhance the treatment response (127). A metastatic PCa antibody-drug conjugate (ADC) using B7H3 as a cell surface marker has also shown good therapeutic effects *in vivo* (128).
2. Histone lysine-specific demethylase 1 (LSD1): LSD1 is an epigenetic regulatory enzyme that plays critical roles in cancers, particularly in tumor cell proliferation, differentiation, and immune escape. LSD1 is an essential companion for FOXA2 to open the chromatin structure. Targeting LSD1 is a potential therapeutic strategy for the treatment of RB1-deficient CRPC and may also slow the transition of CRPC to NEPC to some extent (70, 129). CC-90011 (also known as avadomide) is a selective LSD1 inhibitor developed by Celgene. It has shown good therapeutic efficacy in patients with neuroendocrine lymphoma, including bronchial neuroendocrine tumors, renal tumors, and paragangliomas (130, 131). A phase I clinical trial of CC-90011 in prostate cancer is ongoing, and the findings are being tested in mCRPC patients who fail enzalutamide treatment, although the results are not yet clear (Clinical Trials.gov ID: NCT04628988; Registration Date: 2020-10-27). JBI-802 is a novel dual-target epigenetic modulator that exerts anticancer effects by simultaneously inhibiting both LSD1 and HDAC (histone deacetylase). Clinical studies evaluating this drug in NEPC and other advanced solid tumors are ongoing (Clinical Trials.gov ID: NCT05268666; Registration Date: 2022-02-11). Bomedemstat is an LSD1 inhibitor used for the treatment of primary thrombocythemia and other hematologic diseases, as well as malignancies and small cell lung cancer (SCLC) (Clinical Trials.gov ID: NCT05191797; Registration Date: 2021-12-29). In prostate cancer, bomedemstat has also shown therapeutic potential for several advanced CRPC models and NEPC xenografts (132).
3. Histone deacetylase (HDAC): Vorinostat (also known as SAHA) is an HDAC inhibitor originally approved for the treatment of cutaneous T-cell lymphoma (133). It has shown potential efficacy in various cancers, including prostate cancer, by regulating epigenetics and restoring the expression of tumor suppressor genes. A phase II trial has studied its effect in treating patients with progressive metastatic prostate cancer and evaluated its safety (Clinical Trials.gov ID: NCT00330161; Registration Date: 2006-05-25).
4. Poly (ADP–ribose) polymerase (PARP): The PARP inhibitor talazoparib is widely used to treat cancer patients with DNA repair gene defects (such as mutations in *BRCA1/2)*, including breast, ovarian, and prostate cancers (134). Studies have shown that the PARP inhibitors olaparib (OLA) and talazoparib (TALA) can inhibit the growth of NED and NEPC in NEPC cell lines, organoids, and PDX models (135). Combined treatment with olaparib and CDK4/6 inhibitors (palbociclib or abemaciclib) can effectively inhibit the p-Rb1-E2F1 axis and E2F1 target gene expression, thus leading to the apoptosis of NEPC cells (136).
5. EZH2: Tazemetostat is the first FDA-approved oral small molecule EZH2 inhibitor. It specifically inhibits the methyltransferase activity of EZH2 and reduces the degree of H3K27 methylation to relieve the transcriptional inhibition of tumor suppressor genes. It belongs to the class of epigenetically regulated anticancer drugs. In prostate cancer, especially for CRPC and high-risk prostate cancer, several clinical trials of tazemetostat are ongoing. In the phase Ib/II trial of tazemetostat combined with enzalutamide or abiraterone/prednisone for mCRPC, the disease control rate (DCR) was 47% (Clinical Trials.gov ID: NCT04179864; Registration Date: 2019-11-14). Phase Ia/Ib clinical trials of the PARP inhibitor talazoparib combined with tazemetostat in mCRPC are also ongoing (Clinical Trials.gov ID: NCT04846478; Registration Date: 2021-04-13). CPI-1205, another oral small molecule EZH2 inhibitor, selectively binds to EZH2. In a clinical trial of CPI-1205 combined with enzalutamide or abiraterone/prednisone for the treatment of mCRPC, preliminary results revealed that this combination significantly promoted tumor shrinkage and reduced PSA levels in patients (Clinical Trials.gov ID: NCT03480646; Registration Date: 2018-03-07). In an open-label phase Ib/II study, 47 patients with CRPC-NE received the EZH2 inhibitor mevrometostat, with a median follow-up of 9.7 months. The results revealed that the median radiographic progression-free survival (rPFS) was 17.0 months (95% CI 6.3–NE); 14.9% of patients achieved a PSA50 response; among 22 patients with measurable lesions at baseline, the objective response rate was 27.3%; and the geometric mean reduction in H3K27me3 levels in matched tumor biopsies was 75%. Treatment was well tolerated, with 19.1% of patients discontinuing treatment due to adverse events, and there were no treatment-related deaths (Clinical Trials.gov ID: NCT03460977; Registration Date: 2018-02-12).
6. AURKA: Danusertib (formerly known as PHA-739358) is one of the first Aurora kinase inhibitors to enter clinical trials. It is an ATP-competitive small molecule that inhibits Aurora A, B, and C kinases. Danusertib also inhibits various receptor tyrosine kinases, such as Abl, Ret, FGFR-1, and TrkA, which are involved in the pathogenesis of several malignancies (137). Neuroendocrine differentiation (NED) signaling, driven by the MYCN-PARP-DNA damage response (DDR), is present in CRPC-Adeno and CRPC-Neuro models, and N-MYC stabilization is a key function of AURKA (95). The application of the AURKA inhibitor PHA739358 and the PARP inhibitor OLA can significantly inhibit the progression of NEPC (138). Like danusertib, alisertib (MLN8237) also inhibits N-MYC signaling and tumor growth by inhibiting the interaction between N-MYC and its stabilizing factor, Aurora-A, and is currently used to treat SCLC. In another clinical trial for advanced prostate cancer, although the study did not meet its primary endpoint, some patients achieved significant clinical benefit from alisertib (139). AK1 (LY3295668) has higher specificity and fewer side effects than the other two inhibitors. In both small cell lung cancer and triple-negative breast cancer, AK1 has a “synthetic lethal” effect on RB1-deficient tumors (96). In phase I/II clinical trials, AK1 has shown good antitumor activity and safety (Clinical Trials.gov ID: NCT03092934; Registration Date: 2017-03-22).

### 6.2 Targeting NE-related transcription factors

**Figure 5:**
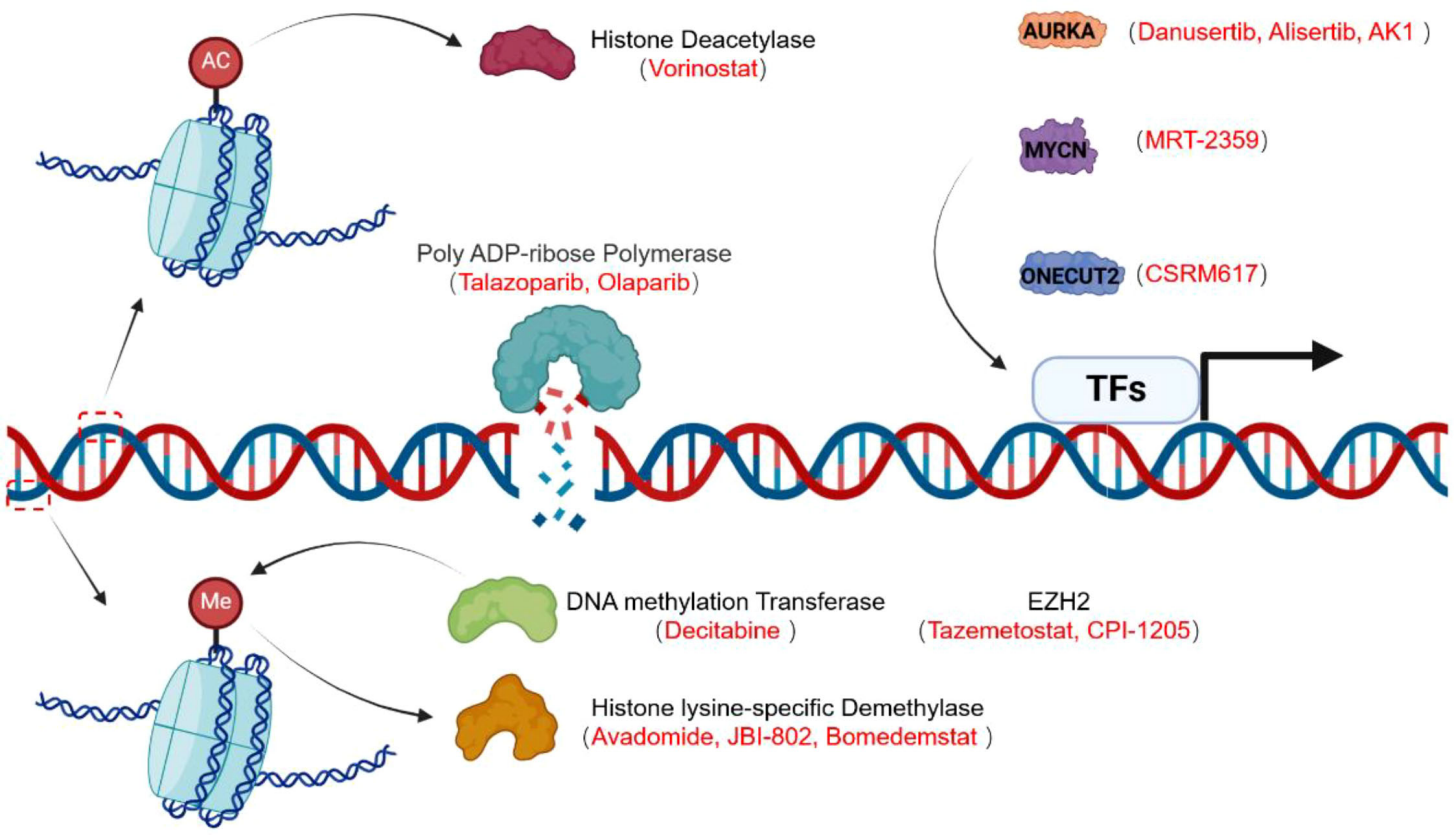
Epigenetic and transcription factor-targeting drugs for the treatment of NEPC. Currently reported targeted drugs for the treatment of NEPC include DNA methyltransferase inhibitors; histone lysine-specific demethylase inhibitors; histone deacetylase inhibitors; PARP inhibitors; and drugs that target MYCN, AURKA, EZH2, and ONECUT2.

1. MYCN: MRT-2359 is an orally bioavailable molecular glue degrader that targets the key protein synthesis regulator GSPT1. It recruits the E3 ligase cereblon (CRBN), leading to subsequent proteasomal degradation. In this way, it selectively induces apoptosis in translationally addicted cells, especially those addicted to Myc-driven protein translation (140). Oral administration of MRT-2359 in non-small cell lung cancer (NSCLC) xenografts and patient-derived xenografts (PDXs) with high N-Myc expression leads to the complete degradation of GSPT1 within tumors, accompanied by a reduction in N-Myc protein levels and resulting in tumor regression. In contrast, MRT-2359 showed limited or no activity in NSCLC models with low N-Myc expression, further confirming the selective vulnerability of Myc-driven tumors to GSPT1 degradation (140). This drug development strategy, based on molecular glues, is becoming a leading direction in cancer treatment, especially with respect to malignant behaviors related to N-MYC overexpression in neuroendocrine carcinoma. MRT-2359 has been approved by the FDA and is undergoing phase I/II clinical trials for lung cancer and neuroendocrine tumors (Clinical Trials.gov ID: NCT05546268; Registration Date: 2022-09-16). Notably, AURKA was identified as a directly associated and indispensable node of MYCN, suggesting that targeting MYCN may also affect the protein function of AURKA (141).
2. ONECUT2: As an important transcription factor driving lineage plasticity changes, the ONECUT2 inhibitor CSRM617 has been shown to suppress CRPC metastases in xenograft models (49) and can also block enzalutamide-induced lineage plasticity (71) (**Figure 5**).

## 7 Immunotherapy

It is well known that prostate cancer is a “cold” tumor, meaning that it responds poorly to immunotherapy. The underlying reason for this is its unique immunological features. The immune genomic landscape of prostate cancer has the following characteristics: (1) Low somatic mutation burden: A significant feature of the immune landscape of prostate cancer is its relatively low somatic mutation burden, which results in fewer neoantigens compared to other cancers (142). A lack of tumor neoepitopes is associated with reduced recruitment of immune cells to the tumor site. Fewer tumor-specific epitope–MHC interactions lead to reduced cross-priming of TILs by antigen-presenting cells (APCs). In this case, transformed cells can escape immune cell-mediated elimination and proliferate freely (143). (2) Loss of MHC/HLA expression: MHC class I proteins can present cytoplasmic peptides to T lymphocytes, triggering an immunostimulatory signaling cascade that leads to T-cell proliferation and target cell lysis (144). Loss of MHC class I expression has been demonstrated in metastatic PCa cell lines and clinical specimens (145, 146). (3) IFN1 signal transduction: IFN1 comprises a group of important immunostimulatory cytokines that recognize invading pathogens through direct binding to their extracellular receptors or innate pattern recognition receptors (147). IFN1 can establish an effective antitumor immune response by promoting cytokine and chemokine production, increasing the expression of immune costimulatory molecules, activating adaptive immune cells, and promoting CTL-mediated killing (148). PTEN loss is a well-defined molecular mutation in PCa. Some studies suggest that the immune regulatory function of PTEN is mediated through regulating the activation of the IFN1 pathway in certain tumors, although this needs further verification in PCa (149). In addition to PTEN, the transcription factors STAT1 and STAT3 drive IFN-1 signal transduction by activating the transcription of various interferon-stimulated genes, which are crucial for IFN-mediated immune function (150). Compared with PTEN loss alone, the combined loss of STAT3 and PTEN can accelerate tumor progression and metastasis via the ARF-MDM2-p53 pathway in mice (151). These findings indicate that the effect of immunotherapy in NEPC is still suboptimal because of the dual impact of inherent genetic characteristics and the absence of surface immune activation signals.

## 8 Conclusions

With the application of organoids, single-cell sequencing, and various sequencing methods, the evolution and progression of NEPC have become increasingly clear. Transcription factors and epigenetic regulation are widely accepted as the driving forces behind PCa transdifferentiation. Based on these findings, an increasing number of epigenetic regulatory elements and inhibitors related to NED transcription factors have been developed, and related clinical studies have been rapidly conducted. Although some studies have achieved preliminary success, the long-term efficacy and potential side effects remain unknown. Additionally, single-cell sequencing revealed that tumor tissues can be divided into multiple subpopulations based on molecular expression differences, and the transcription factors governing cellular characteristics also vary significantly across these subpopulations. This heterogeneity, driven by molecular factors, is particularly significant in NEPC. Taking ASCL1 and NEUROD1 as examples, their expression is mutually exclusive in NEPC, existing as independent subpopulations (40, 152–154). Such diversity in expression patterns directly determines the pathological characteristics, cellular morphology, and clinical behavior of tumors. At the histological level, ASCL1+ cells tend to grow in a sheet-like pattern, with spindle-shaped or fusiform morphology, relatively abundant cytoplasm, and finely granular nuclear chromatin. These morphological features resemble those of classic neuroendocrine tumors but are associated with a more aggressive phenotype. In contrast to the ASCL1 subtype, NEUROD1-driven NEPC cells exhibit distinctly different pathological morphology. NEUROD1+ cells tend to grow in small cell clusters, with smaller cell size, a high nuclear-cytoplasmic ratio, coarsely granular or “salt-and-pepper”–like nuclear chromatin, and inconspicuous nucleoli. This morphology is highly similar to that of small cell lung cancer (SCLC) (40). From the perspective of molecular regulatory networks, the downstream regulatory network of NEUROD1 partially overlaps with that of ASCL1 but also shows substantial differences. ChIP-seq analysis reveals strong enrichment of the NKX2 motif at ASCL1-specific binding sites, whereas the EBF and LHX motifs are enriched at NEUROD1-specific sites; both transcription factors share the NFIB motif (40). In terms of therapeutic response, ASCL1-driven NEPC cells are more sensitive to DLL3-targeted therapies (e.g., tarlatamab), as ASCL1 serves as a key regulator of DLL3 expression (84). In comparison, NEUROD1+ cells also show a better response to Aurora A kinase inhibitors (e.g., alisertib) than ASCL1+ cells, which may be related to the synergistic interaction between NEUROD1 and N-Myc (155). Furthermore, single-cell sequencing analyses of clinical specimens have revealed a more complex expression landscape. Using an RPM (Rb1/Trp53/Myc) prostate organoid allograft model, Park et al. reported that approximately 70% of INSM1 cells were ASCL1 , 15% were NEUROD1 , and 2% were double-positive for ASCL1 and NEUROD1. In addition, 13% of INSM1 cells expressed neither ASCL1 nor NEUROD1, suggesting the existence of potentially uncharacterized neuroendocrine subtypes (152). From spontaneous tumor-forming NEPC mouse models, it can be inferred that the development of NEPC may reflect a population advantage, where the growth advantage of different subpopulations, represented by distinct driver molecules, manifests as PCa transdifferentiation (19). Targeting a single key driver factor may lead to the dominant growth of another subpopulation. Therefore, although significant progress has been made in targeting transcription factors, we must carefully assess the benefits and drawbacks of this therapeutic approach.

In addition to epigenetic and transcription factor-targeting strategies, novel targets on NEPC surface antigens have been increasingly identified and are advancing into preclinical or early clinical research. DLL3, one of the most extensively studied targets to date, is highly expressed in NEPC but nearly absent in normal tissues, demonstrating excellent tumor specificity. Various strategies targeting DLL3, including antibody–drug conjugates (e.g., SC16LD6.5), bispecific T-cell engagers (e.g., AMG 757), radioimmunotherapy (e.g., 177Lu-DTPA-SC16), and immuno-PET molecular imaging ([89Zr]Zr-DFO-SC16.56), have demonstrated good efficacy and imaging specificity in NEPC models, with some already entering clinical validation phases (156–159). Another potential target, CEACAM5, is highly expressed in NECCs and regulated by the transcription factor ASCL1; the ADC drug lanuzumab govitecan has shown significant antitumor activity in chemotherapy-resistant models (84). Notably, CD46, a membrane protein highly expressed in both adenocarcinoma and NEPC but with limited expression in normal tissues, has also been identified as an ADC target, inducing complete remission in CRPC xenograft models and holding potential for cross-subtype therapy (160).

For NEPC, future targeted approaches should simultaneously target multiple NE-related transcription factors or inhibit the chromatin accessibility of NE-related genes to comprehensively block NE differentiation. Based on current research, molecular degradation strategies, such as the use of molecular glues and PROTACs, appear to be promising options.
